# Maxillofacial Surgery Within the Archive of the Greek Surgeon of the 19th Century Theodoros Aretaios (1829–1893)

**DOI:** 10.7759/cureus.74195

**Published:** 2024-11-21

**Authors:** George Filippidis, Georgia Fragou, Irina Noskova, Evangelia Mourellou, Dimitrios Zisiadis, Konstantinos Laios

**Affiliations:** 1 Medical School, National and Kapodistrian University of Athens, School of Medicine, Athens, GRC; 2 Faculty of Nursing, National and Kapodistrian University of Athens, Athens, GRC; 3 Social Medicine, University of Crete, Heraklion, GRC; 4 Surgery, National and Kapodistrian University of Athens, School of Medicine, Athens, GRC

**Keywords:** cleft palate, facial oncology, hellenic surgery, parodit gland, scirrhus

## Abstract

One of the most important figures of the Hellenic surgery of the 19^th^ century, professor of the Othonian University of Athens, Theodoros Aretaios (1829-1893), portrays in his personal archives a series of surgical operations in the field of maxillofacial surgery. During his career, he operated the following surgical diseases, these are adenosarcomas or inosarcomas of the parotid region, osteofibroma of the sinus antrum, osteosarcomas of the upper and lower jaw, and lycostoma (cleft palate). He was able to perform radical enucleations of the tumorous masses. He reconstructed the area as a plastic surgeon. He had great surgical speed. In addition, the use of modern surgical tools, his ability to drain with catheters surgical wounds, and his skills to manipulate soft tissues, bones, and teeth, resulted in satisfactory outcomes. This historical vignette, by conducting documentary research in Theodoros Aretaios Archives, kept in the National Library of Greece, unveils one more surgical edge of a great surgeon.

## Introduction and background

**Figure 1:**
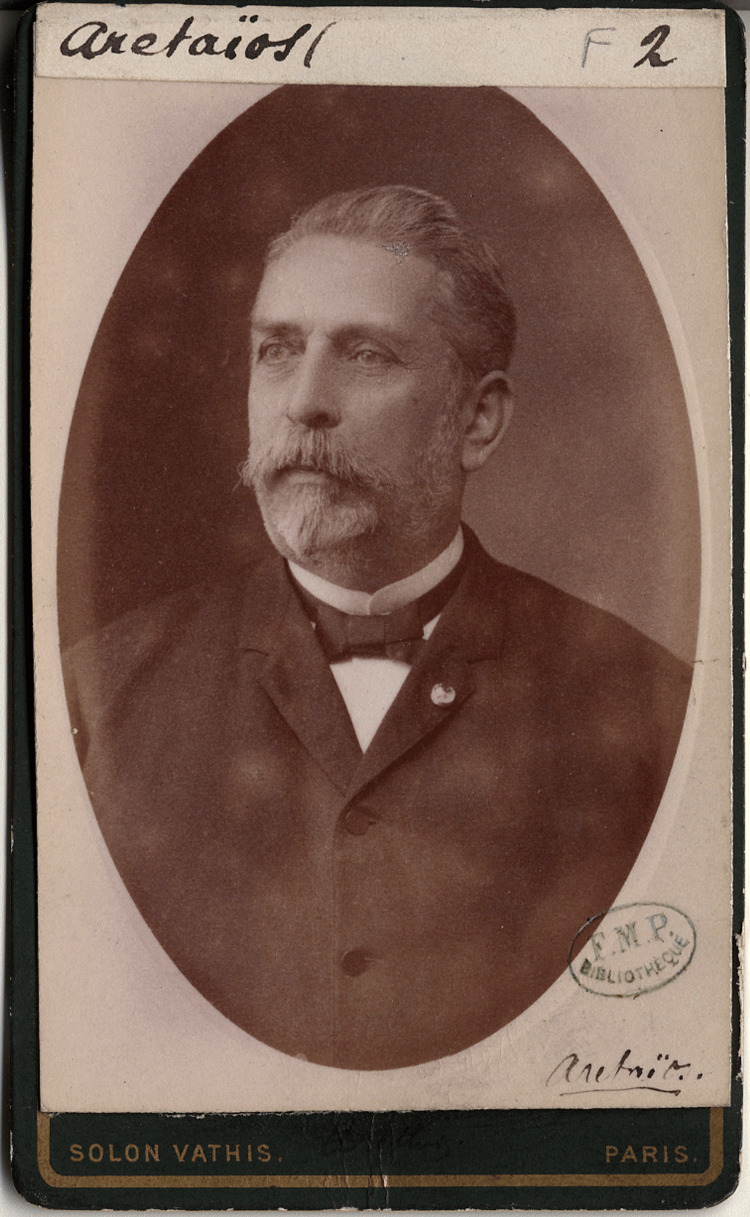
Theodoros Aretaios, by Solon Vathis, photographed in Paris, date unknown. Holding no rights

Interventions related to the field of maxillofacial surgery, as performed in the 19th century, involved surgical excisions of malignant tumors primarily in the parotid gland, procedures for managing malignant transformation, particularly in the form of osteosarcoma of the jawbones, as well as attempts made for the plastic reconstruction of cleft palate. Since this field required numerous delicate maneuvers, it should be emphasized that references to related surgeries found in the archives of Theodoros Aretaios (Greek: Θεόδωρος Αρεταίος, 1829-1893) (Figure 1) indicate that the surgical approaches were performed with amputative characteristics. However, the extent of the surgery without causing neurological damage, or the reports of healing, allow us to hypothesize that either these procedures were not performed with precise oncological characteristics, or that the references to the restoration of health concerned only a short observation period [1].

This historical vignette reports Aretaios’ skills in maxillofacial surgery, celebrating one of the most important figures of 19th-century Hellenic surgery. His operations were considered pioneering, attracting global interest. Apart from his skills, his surgical procedures were characterized by speedy and precise outcomes [1]. Theodoros Aretaios was born in Nauplion, originally named Konstantinidis, changing later his surname to Aretaios after a royal decree from the Hellenic Kingdom [2]. Theodoros started his studies in medicine at the University of Athens in 1849, being further educated in Berlin, Vienna, and Paris. Upon his return to Greece, he was appointed as head physician of the surgical department of the newly founded hospital in Athens, Astyclinic. In 1863, he was elected as the Professor of the Artistic Anatomy and Epidesmologia in the School of Medicine of the Othonion University of Athens, later becoming Regular Professor of Surgery and Dean of the School [2-3]. He wrote a series of treatises “On Surgery” and “Surgical Pathology” [4-5]. After conducting documentary research in the Theodoros Aretaios Archive kept in the Manuscripts Department of the National Library of Greece, a series of causes related to maxillofacial surgery testify both knowledge and diversity of cases being treated during Aretaios era in the surgical theaters of the University of Athens in 19th century.

## Review

Tumorous masses

Since 1876, there are references to the excision of adenocarcinoma in the right parotid gland in a 30-year-old female patient, as well as an uncharacterized fibrosarcoma in the parotid gland of an 18-year-old male patient [6]. In the female patient, the appearance of the formation was noted to have occurred seven years earlier, and its sudden increase in size to that of a walnut was associated with her suffering from typhoid fever. It was stated that during the excision of the formation, which was characterized as adenosarcoma, a portion of the parotid gland was also removed, along with branches of the facial nerve, which was recognized as the cause of the postoperative cheek and eyelid paresis the patient exhibited, a description reminiscent of facial nerve paresis. Although it is mentioned at the end of the report that the healing process was progressing, no further information is provided regarding the course of the paresis. In the case of the young man, the formation was the size of an egg and occupied the area beneath the ear down to the angle of the jaw. The formation had a textured surface, and the procedure involved its relatively easy excision without additional injuries.

In 1882, a 16-year-old male patient with a sizable formation was treated in the same clinic, characterized as an osteofibroma of the left maxillary sinus that occupied the corresponding upper jaw [7]. In the medical history of the patient, the appearance of the tumor was linked to an accident he had at the age of 11 when he fell and injured his left zygomatic bone, from which the progressive growth of the tumor was noted to have developed after one year. Theodoros Aretaios performed two incisions on the skin: a vertical one from the inner canthus to the upper lip, and a transverse incision along the suborbital ridge. He penetrated the soft tissues of the area above the tumor and exposed it, discovering that it had needle-like prominence. Next, with two crescent-shaped incisions, he incised the surface of the tumor and peeled it away. Then, using a narrow saw, he performed a resection of the upper jaw, which had been infiltrated by the tumor from its nasal projection, its inferior surface, and the body of the zygomatic bone, penetrating through the suborbital fissure and into the left nasal cavity, where he incised the palatine projection. Ultimately, he removed the prepared segment of the jaw along with the lower wings of the sphenoid bone, flattening the stump with the use of bone scissors. The postoperative course was smooth, and gradually, the defect from the bone resection was covered by the development of fleshy tissue. It was noted that communication between the nasal cavity and the maxillary sinus was established due to the surgery.

In 1884, we came across a case involving a 60-year-old male patient diagnosed with osteosarcoma of the upper jaw [8]. The initial symptom was pain in the left cheek and the teeth of the upper jaw, which led to the extraction of the primarily painful tooth. Although this procedure provided temporary relief, the pain recurred, and a tumor the size of a pea appeared on the suborbital ridge. This tumor gradually grew to the size of a goose egg and occupied the area from the orbit of the left eye to the temple and the cheek. Indeed, the tumor had occupied the orbit and displaced the eye backward, making it difficult to see, and it could only be viewed with difficulty through the use of fingers. The lower boundary of the tumor was the alveolar projection, leading Theodoros Aretaios to conclude that the tumor originated from the maxillary sinus. He proceeded to excise the tumor while simultaneously performing a resection of the upper jaw on the left side, initially making vertical incisions from the inner corner of the eye to the orbital rim, and then horizontally from the end of the orbit to the temple. The tumor was excised along with the resection of the upper jaw on the left using a narrow saw, and it was noted that the entire operation lasted three-quarters of an hour, during which the patient exhibited great bravery. During the wound dressings, it was noted that the eye was damaged due to pressure from the tumor, but there was no mention of its removal.

After 6 years the patient suffered a recurrence. The new tumor occupied the entire size of the cavity formated after the bone excision, but it was now inoperable because of the large size of the recurrence protruding from the cavity, extending significantly forward without any signs of the eyeball, which likely had been removed during the postoperative period.

In a male 73-year-old patient in 1892, the appearance of a mass was reported, characterized as an osteosarcoma in the middle of the lower jaw [9]. It is stated that the patient had lost the teeth of the lower jaw a long time ago, while the mass had grown to the size of a goose egg, which led the patient to seek help. Theodoros Aretaios removed the excess skin from the jaw and, after producing it from the soft tissues and encircling the median fold of the tongue in order, as stated by the archive, to prevent the swallowing of the tongue, used a saw, the type of which is not specified, to excise from the base of the mouth of the affected part of the lower jaw to the healthy tissues. The skin was initially sutured, and it is noted that the bone healed normally to a large extent. Although this case was deemed treatable, a similarly unfortunate outcome occurred in 1886 with a 60-year-old male patient again suffering from osteosarcoma of the lower jaw [10]. The malignant transformation had affected the right half of the lower jaw, leading to the excision of the affected bone. Theodoros Aretaios placed two drainage tubes at each end of the surgical wound, and for the first time in such a case, it is reported that a rubber tube was placed from the nostril to the pharynx for food intake. Despite this, the patient would succumb to sepsis on the fifth postoperative day, despite the wound dressings and antiseptic rinses he underwent.

Cleft palate

The pathology of cleft palate also concerned Theodoros Aretaios, who attempted to provide a surgical solution for his 23-year-old male patient in 1886 by performing the osteoplastic technique of Langenbeck [11]. This patient presented with a unilateral division of the osteoid hard palate, the alveolar projection, and the lip toward the left, with the division of the soft palate located centrally. The gap he exhibited, which also caused him to have a harelip, had further affected his speech, as it was stated that “ the patient’s speech is completely unclear.” The surgery he performed was partially successful, as he himself stated. He described that because the gap was asymmetric, he created a flap on the left side, while on the right side, only the periosteum was detached from the mucosa of the palate. The gap in the osteoid hard palate was divided into two openings, one anterior and oval on the left, and a smaller posterior one. The anterior edge of the flap was narrow due to the defect in the alveolar projection, which confirmed the surgeon’s initial fears of necrosis in the anterior part, which occurred on the fourth postoperative day, as did the median suture. On the 10th postoperative day, when enough tissue had developed at the edges of the debriding, a new suture was performed on the right edge of the cleft, resulting in a complete union of the soft palate. However, the patient’s speech did not improve. He expressed the thought that perhaps the placement of an artificial palate would improve speech, although he was not certain about this, only that the artificial palate would certainly benefit the interruption of communication between the nasal cavity and the mouth, as well as allowing for perfect swallowing of food. The patient had previously undergone surgery for the treatment of his harelip, but without success, which is why now, approximately three months after the surgery, he would successfully correct it this time, as stated in the report of the incident, which concludes his description.

Discussion

**Figure 2:**
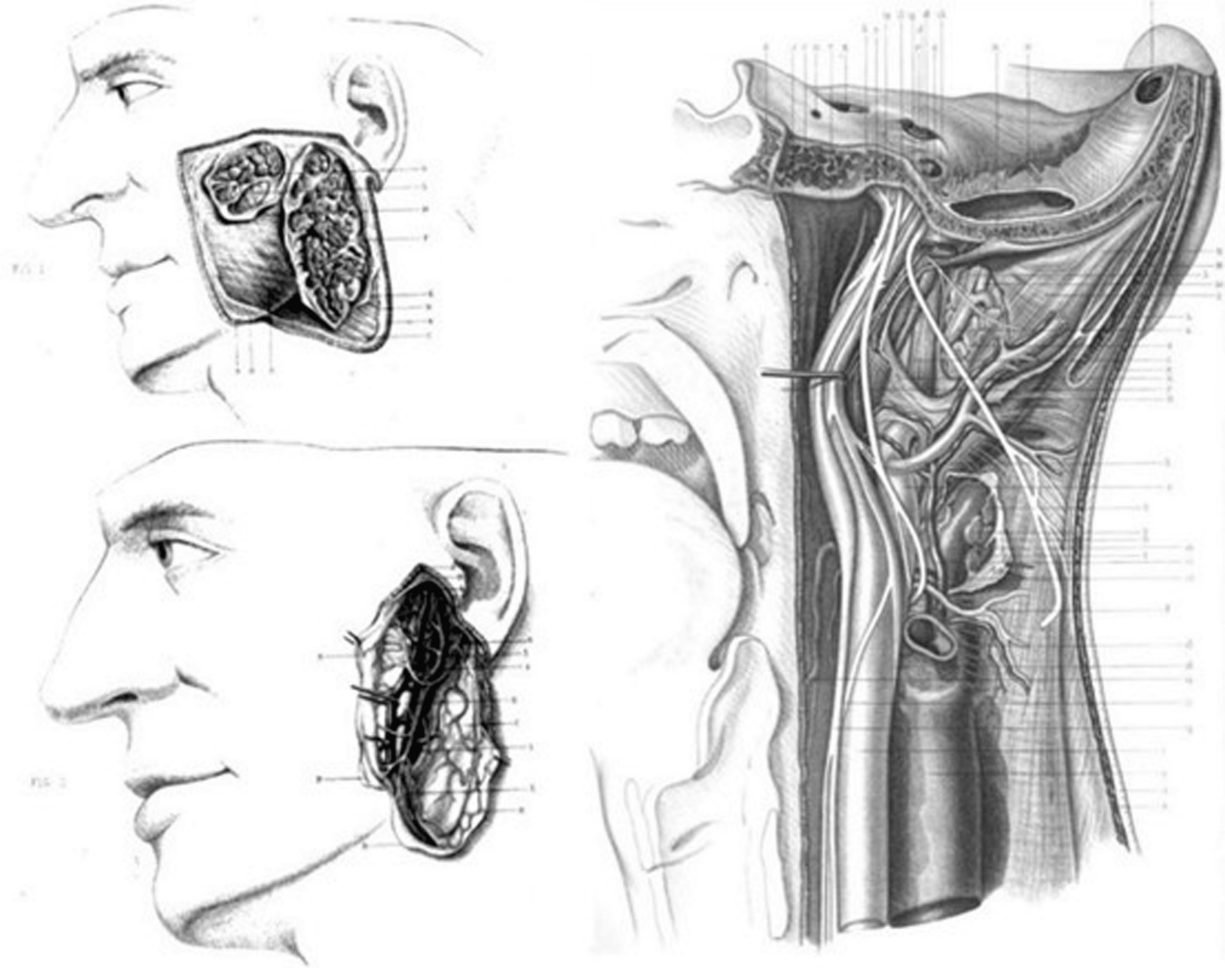
Parotid region and the deep relations of the parotid region by Bruno Jacques Béraud in his work Atlas of surgical and topographical anatomy, 1867. That is superficial and deeper lobes as well as the connection to the facial artery. Image credit: Open Government License, ODC-BY, and CC-BY 2.0 terms

The advancements produced during the 19th century as the use of anesthesia with ether, introduced by Crawford Long and Charles Jackson in 1846 in Massachusetts, and the antisepsis protocol by Lister in 1867, offered surgeons the possibility of more invasive operations, demonstrating the surgical extraction of malignant masses and amputations combined with reconstruction of the tissues of the area [12-13]. In the late 19th century cocaine hydrochloride was introduced for local anesthesia. Enucleation of the tumorous entities was advocated, with extreme caution of preservation of the facial nerve and minimal damage to the parotid plexus. Morbid complications of facial paralysis or other neuro-lesions of the area were in place. As the capsule of the tumor usually had pseudopodia or satellite masses, recurrence could be observed, yet surgeons couldn’t always define the reason why [14]. Surgery in the area of the parotid plexus began around 1826 and surgeons attempting to perform such operations were characterized as bold. German Lorenz Heister (1683-1758) and Théophile de Bordeu (1722-1776) were among the pioneers. Although noted that anatomical obstacles and the almost inaccessible situation of the gland made the operations rather difficult. American George McLellan (1796-1847) had performed 11 operations near the parotid plexus in 1838 [15]. Topographical anatomy of the area was studied since the 17th century by anatomist and bishop Niels Steensen (1638-1686) [16-17], while surgical atlases of the 19th century depicted the region in detail (Figure 2) [18]. Facial tumors, especially all those resembling scirrhus, were to be radically excised [19]. Aretaios followed the up-to-date methods of the era, performing wide excisions of the tumorous masses with proficient knowledge of the topographical anatomy, exceedingly sometimes the borders of a general surgeon into the grounds of a specialized maxillofacial or dental surgeon.

**Figure 3:**
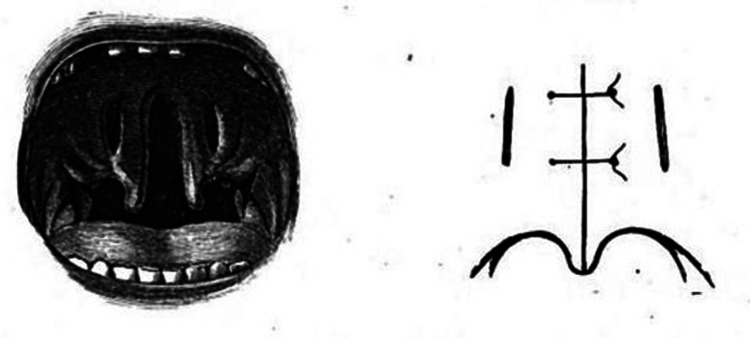
Cleft palate and sutures by Francis Mason in his work “On Harelip and Cleft Palate” in 1877. Cleft palate on the left and the scheme of the sutures on the right. Image credit: Open Government License, ODC-BY, and CC-BY 2.0 terms.

Congenital fissures of the palate assume a variety of forms and in some cases the vomer may be placed free and exactly in the median line so that a probe may pass into the nasal cavity, as books of surgery noted and sutures were considered the gold standard (Figure 3). In case of hard palate deficiency, the gap could be filled by mucous membrane alone, while some proposed the use of a silver plate. Since 1820, American surgeon John Collins Warren (1778-1856) performed closures of the soft palate, while Wenzel Krimer (1795-1834) four years later started closing the hard palate. The age between 14 and 16 years, was considered the best to perform a cleft palate correction. German Christian Albert Theodor Billroth (1829-1894) proposed young age performing an operation on a six-month child [20]. Aretaios performed the operation on an elderly patient, aged 23, with some complications, considering an artificial palate without mentioning the material. He had reconstructed cleft lip during a following operation to improve vocal quality.

## Conclusions

In all cases, Aretaios masterly maneuvered around the anatomical entities of the face, exploited all available tissues, drained the operated area, and contrived the desired outcome. In an era of progressive surgery and continuous advancements, Aretaios remained in vogue to offer the best medical service for his patients. The successful management and the daring operations were similar to those being performed around Europe. His skills gained him a place among the best and the survey of his documents always raised great interest among the researchers of the history of medicine.
